# A Novel Transient Fault Current Sensor Based on the PCB Rogowski Coil for Overhead Transmission Lines

**DOI:** 10.3390/s16050742

**Published:** 2016-05-21

**Authors:** Yadong Liu, Xiaolei Xie, Yue Hu, Yong Qian, Gehao Sheng, Xiuchen Jiang

**Affiliations:** School of Electronic Information and Electrical Engineering, Shanghai Jiao Tong University, Shanghai 200240, China; xiexiaolei0116@126.com (X.X.); yuehu@sjtu.edu.cn (Y.H.); qian_yong@sjtu.edu.cn (Y.Q.); shenghe@sjtu.edu.cn (G.S.); xcjiang@sjtu.edu.cn (X.J.)

**Keywords:** overhead transmission line, fault current, PCB Rogowski coil, differential winding, open structure, high-frequency transient fault current

## Abstract

The accurate detection of high-frequency transient fault currents in overhead transmission lines is the basis of malfunction detection and diagnosis. This paper proposes a novel differential winding printed circuit board (PCB) Rogowski coil for the detection of transient fault currents in overhead transmission lines. The interference mechanism of the sensor surrounding the overhead transmission line is analyzed and the guideline for the interference elimination is obtained, and then a differential winding printed circuit board (PCB) Rogowski coil is proposed, where the branch and return line of the PCB coil were designed to be strictly symmetrical by using a joining structure of two semi-rings and collinear twisted pair differential windings in each semi-ring. A serial test is conducted, including the frequency response, linearity, and anti-interference performance as well as a comparison with commercial sensors. Results show that a PCB Rogowski coil has good linearity and resistance to various external magnetic field interferences, thus enabling it to be widely applied in fault-current-collecting devices.

## 1. Introduction

The overhead transmission line is an important part of the power supply network. However, long-distance overhead transmission lines are exposed to complicated environments and are easily damaged by lightning strikes and foreign matter. This issue can cause a certain amount of transient current, which contains considerable fault information and is the basis of fault location and diagnosis (e.g., transient protection, fault analysis, and travelling wave fault location).

Analyses based on high-frequency components such as high-frequency protection, fault diagnosis, travelling wave fault location, *etc.*, are more popular than ever [1,2]. Therefore, the real-time transient current monitoring of overhead transmission lines is the basis for fault detection and diagnosis [3]. Existing current-measuring devices include electromagnetic current sensors [4], Hall current sensors [5], optical current sensors [6], and Rogowski coils.

Traditional electromagnetic current sensors cannot accurately measure high Ampere currents because of the saturation problem of the magnetic core. A high-frequency transient fault current is also difficult to measure because of the working frequency limit of the magnetic core. The Hall current sensor is significantly influenced by the environmental temperature and has poor stability, so it is difficult to capture the fault transient current. The newest optical current sensor based on the Faraday rotation effect can measure both transient and direct currents. Although this new sensor is anticipated to be the direction of current measurements in the future, it is difficult to use in power systems in the short run because of its poor anti-interference properties and high cost.

A Rogowski coil is the optimum measuring method for high-frequency transient currents at the moment. However, parameter consistency is hard to guarantee because it is impossible for two random Rogowski coils to have uniform windings and the same cross-section between each turn, so as a result, a Rogowski coil can only be used after calibrations by an integrator [7]. What’s more, the commercial Rogowski coils represented by Pearson (Palo Alto, CA, USA), PEM (Nottingham, UK), and Rocoil (Harrogate, UK) are mainly used as the sensor of precise instruments are costly, thereby limiting their wide applications.

The printed circuit board (PCB) Rogowski coil has attracted increasing attention recently because of its characteristics of high accuracy, small volume, low cost, and easy mass production [8,9,10,11,12,13]. Considering that overhead transmission lines cannot be out of service once they are activated an open structure sensor shall be designed to collect the transient fault currents of overhead transmission lines. However, the poor electromagnetic environment surrounding overhead transmission lines also requires a PCB coil to have high anti-interference capability. In this paper, a differential winding PCB Rogowski coil was designed for open structure [14]. A joint structure of two semi-rings, as well as collinear twisted pair differential windings in each semi-ring, were employed to eliminate vertical magnetic field interference, moreover, the symmetric wiring of the PCB was used to ensure symmetrical windings and improve its parallel anti-interference characteristics. This open structure PCB sensor can realize hotline installation, with high practicability. As a result, the designed PCB sensor could facilitate the low-cost and large-scale production of transient fault current sensors.

## 2. The Principle of the Rogowski Coil

**Figure 1:**
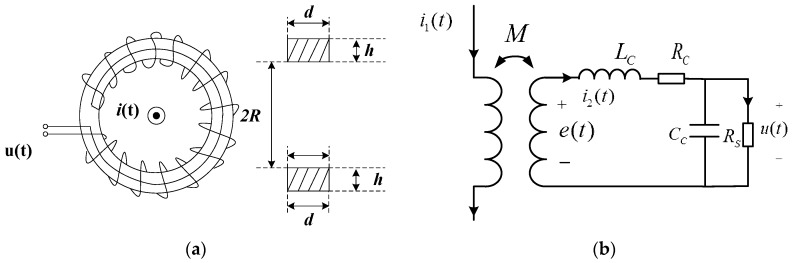
Principle of a Rogowski Coil. (**a**) Structure Diagram of a Rogowski coil; (**b**) Equivalent circuit of a Rogowski coil.

The Rogowski coil consists of two parts (Figure 1a). One is the bobbin made of non-permeability magnetic materials (the material permeability can be viewed approximately equal to the permeability of vacuum μ_0_). The bobbin has a uniform texture, and its cross section is generally rectangular or round. The other part is wound up on the frame tightly and evenly. The Rogowski coil induces a voltage signal that represents the testing current when flux changes.

If the terminal resistance is *R_s_*, the equivalent circuit of a Rogowski coil can be shown as Figure 1b, and the output of Rogowski coil can be [15,16]:

(1) $$e\left(t\right)=M\frac{di_{1}\left(t\right)}{dt}=L_{c}\frac{di_{2}\left(t\right)}{dt}+R_{c}i_{2}(t)+u\left(t\right)$$

(2) $$i_{2}\left(t\right)=C_{c}\frac{du\left(t\right)}{dt}+\frac{u(t)}{R_{s}}$$

Combining Equations (1) and (2) yields:

(3) $$M\frac{di_{1}\left(t\right)}{dt}=L_{c}C_{c}\frac{d^{2}u\left(t\right)}{dt^{2}}+\left(\frac{L_{c}}{R_{s}}+R_{c}C_{c}\right)\frac{du\left(t\right)}{dt}+(1+\frac{R_{c}}{R_{s}})u\left(t\right)$$

where *C_c_* is the distributed capacitance, *R_c_* is the resistance inside the coil, *L_c_* is the self-inductance of coil, and *M* is the mutual inductance of coil. In most cases, *C_c_* is very little and can be omitted, so the output of Rogowski coil can be:

(4) $$e\left(t\right)=L_{c}\frac{di_{2}\left(t\right)}{dt}+(R_{c}+R_{s})i_{2}(t)$$

So when $L_{c}\frac{di_{2}\left(t\right)}{dt}\gg (R_{c}+R_{s})i_{2}(t)$, which means $\omega L_{c}\gg R_{c}+R_{s}$, Equation (1) will be:

(5) $$M\frac{di_{1}\left(t\right)}{dt}\approx L_{c}\frac{di_{2}\left(t\right)}{dt}\Rightarrow Mi_{1}\left(t\right)\approx L_{c}\frac{u\left(t\right)}{R_{s}}$$

As the result the relationship between output voltage and input current is:

(6) $$i_{1}\left(t\right)=\frac{L_{c}}{MR_{s}}u\left(t\right)$$

Output is proportional to the input, and the Rogowski coil is operating in self-integration model, and when $L_{c}\frac{di_{2}\left(t\right)}{dt}\ll (R_{c}+R_{s})i_{2}(t)$, Equation (1) will be:

(7) $$M\frac{di_{1}\left(t\right)}{dt}\approx (R_{c}+R_{s})i_{2}(t)\Rightarrow i_{1}\left(t\right)=\frac{\left(R_{c}+R_{s}\right)}{M}\int \frac{u\left(t\right)}{R_{s}}dt$$

At this time, the input current is equal to the integration of the output voltage, and the Rogowski coil is operating on an external-integration model.

## 3. Analysis on the Interference Sources Surrounding Overhead Transmission Lines

The electromagnetic environment surrounding the overhead transmission line is complicated. Thunder, lightning, discharges, and fault transit currents, except for the power frequency magnetic fields (PFMFs) around overhead transmission lines, will all generate electromagnetic interferences which can influence the measurement of a PCB Rogowski coil.

**Figure 2:**
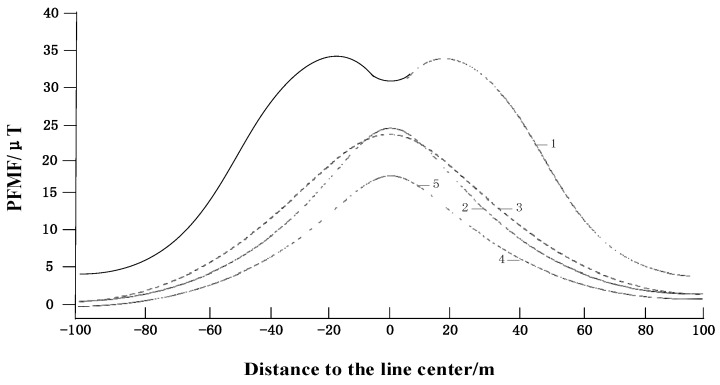
Five PFMF distributions in the UHV overhead transmission line route at 1 m height from the ground. 1—Parallel arrangement of phase conductor; 2—Triangle arrangement of phase conductor; 3—Common-tower double-circuit overhead transmission line; 4—Compact type (10 splits); 5—Compact type (12 splits).

In order to estimate the magnetic field around the overhead transmission lines, [17] calculated the magnetic field with five different conductor configurations. Assuming all the overhead transmission lines were 22 m from the ground, the selected point was 1 m above the ground. The calculated magnetic field distribution when the selected point from the overhead transmission lines is moved to a farther point is shown in Figure 2, which shows that the PFMFs grow large when close to the overhead transmission lines. For the transient fault current sensors installed on overhead transmission lines, the monitoring object is the fault current which is several times larger than the nominal current and will cause much more interference to the transient fault current sensor, hence, the interference from an adjacent conductor is the main interference source for the transient fault current sensor, meanwhile, the interference caused by discharge will affect the sensor at all directions.

### 3.1. Analysis on the Interference of Adjacent Wire

**Figure 3:**
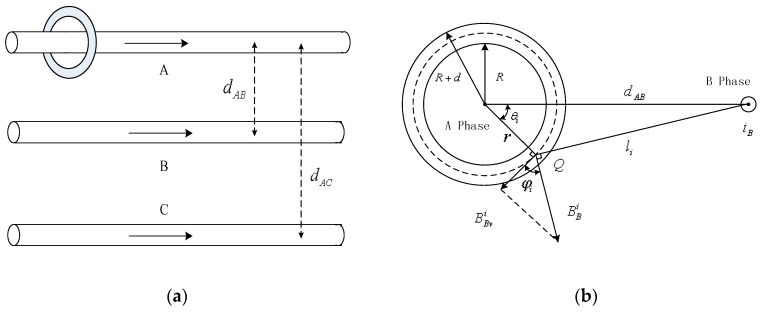
Interference of adjacent conductors. (**a**) Interferences of adjacent phases; (**b**) Effect of the Phase-B magnetic field.

When the Rogowski coil is installed on the overhead transmission line (Phase A), the magnetic fields generated by the adjacent line (Phases B and C) will also couple in the coil and produce an induced electromotive force as shown in Figure 3a. The output of the sensor is the sum of all the induced voltages produced by the corresponding conductors. In order to estimate the interference from the adjacent line, phase B is taken as an example to analyze in Figure 3b.

The distance between Phase-A and Phase-B conductors is *d_AB_* as shown in Figure 3b. If one point Q exists on the *i*th turn of Rogowski coil, which is *r* away from the circle center, and the distance between Phase-B conductor and point Q is *d_AB_*, the angle θ_*i*_ is the included angle between *r* and *d_AB_*.

According to Ampere’s circuit law, the magnetic induction intensity generated by the Phase-B conductor on point Q is on the vertical line to line B_Q_, and only the magnetic induction intensity perpendicular to the coil cross section, *i.e.*, the tangential direction of point Q, has a contribution to the induced electromotive force. The magnetic induction intensity component in the tangential direction of point Q is expressed as follows:

(8) $$B_{Bv}^{i}(t)=B_{B}^{i}(t)\text{cos}\;\varphi_{i}=\frac{\mu_{0}i_{B}(t)}{2\pi l_{i}}\text{cos}\;\varphi_{i}$$

The total turns of a Rogowski coil is *N*. If a Rogowski coil is wound evenly and tightly, the induced electromotive force can be expressed as the following continuous integral form:

(9) $$e(t)=\frac{di_{B}(t)}{dt}\frac{\mu_{0}h}{4\pi }\int_{0}^{2\pi }\frac{N}{2\pi }\text{ln}\frac{{\left(R+d\right)}^{2}+d_{AB}^{2}-2(R+d)d_{AB}\text{cos}\;\varphi }{R^{2}+d_{AB}^{2}-2Rd_{AB}\text{cos}\;\varphi }d\varphi$$

On the basis of numerical calculations with MATLAB, when *d_AB_* > (R+d), *e*(*t*)=0. This result shows that as long as the adjacent conductor is beyond the PCB Rogowski coil and the winding of the PCB Rogowski coil is uniform, the interference from adjacent conductors can be omitted.

### 3.2. Analysis of Vertical External Magnetic Field Interference

**Figure 4:**
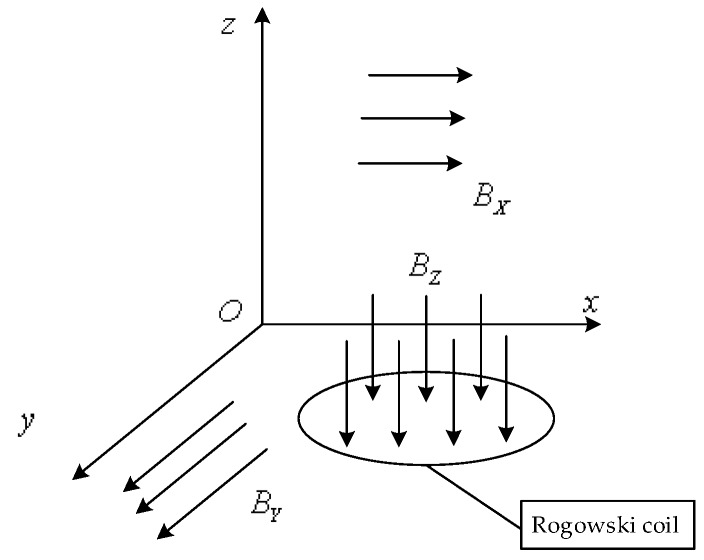
The impact of orthogonal magnetic field.

There are various interfering magnetic fields around the sensor, which can be decomposed into *B_X_*, *B_Y_* parallel to the plane *xOy* where the bobbin is located, and *B_Z_* perpendicular to the plane *xOy*, as shown in Figure 4 The impact of *B_X_* and *B_Y_* on the Rogowski coil is same as that of an adjacent conductor, so only the impact of *B_Z_* needs to be considered.

**Figure 5:**
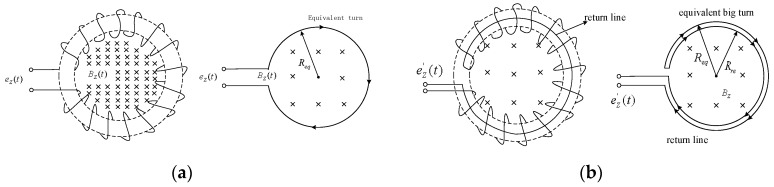
The return line of Rogowski coil. (**a**) Equivalent large turn without return line; (**b**) Rogowski coil with the return line.

*B_z_* is parallel to the cross-section where the Rogowski coil was located, thus, no induced electromotive force will be generated in the winding. However, the winding attaches on the coil bobbin spirally, thereby forming a structure similar to an annular solenoid. After the coil winds around the bobbin for one circle, an equivalent large turn is formed as shown in Figure 5a.

*B_z_* is perpendicular to the plane where large turns are located. When *B_z_* changes with time, the magnetic flux (Ψ_*Z*_) will produce an induced electromotive force (*e_z_*) in the Rogowski coil. Although the large turn has only one turn, its area is often significantly larger than the sectional area of windings. As a result, it will cause a significant error in the measured result. In practice, a return line [18] shall be considered in a practical Rogowski coil to avoid the interference from *B_z_* as shown in Figure 5b. The plane where the return line was located was parallel to plane *xOy*. If the radius of the circle formed by the return line is *R_re_* and the induced electromotive force generated by *B_z_* on the return line is *e_re_*, the final output induced electromotive force *e’_z_* is equal to the sum of *e_re_* and *e_z_*. If *R_re_* is determined equal to the equivalent radius of the large turn (*R_eq_*), *e_re_* is equal to *e_z_* in numerical value but has an opposite direction. This opposite direction can be attributed to the opposite current in the return line to that of the equivalent large turn. Therefore, the effect of the vertical magnetic field on Rogowski coil performance can be effectively eliminated by adding return lines.

## 4. Design of Differential Winding PCB Rogowski Coil

For the fault current sensors installed on the overhead transmission line, the work temperature ranges from −40 °C to 85 °C, so the shape of the bobbin could change with the temperature and cause measurement errors. Meanwhile, the overhead transmission line requires the sensor to be installed without power interruption, so an open sensor structure is indispensable, so the main task to develop a transient fault current sensor based on the PCB Rogowski coil is how to eliminate the errors caused by temperature and interference. 

### 4.1. Material Selection for the Rogowski Coil Bobbin

The coefficient of thermal expansion was considered when selecting the materials for the Rogowski coil bobbin. The coefficients of thermal expansion of some common materials are listed in Table 1 [19]. 

Rubber and polyethylene have significantly high thermal expansion coefficients, which will cause large temperature errors in environments where the working temperature changes greatly. Although ceramics have a significantly low coefficient of thermal expansion, they break easily and have a low rate of finished products. Long-term vibrations on overhead transmission lines and sudden electrodynamic forces on short circuits also occur; these phenomena can destroy the ceramic bobbins easily. As a result, epoxy resin was selected as the base material of our PCB.

### 4.2. Differential Winding of the Rogowski Coil

**Figure 6:**
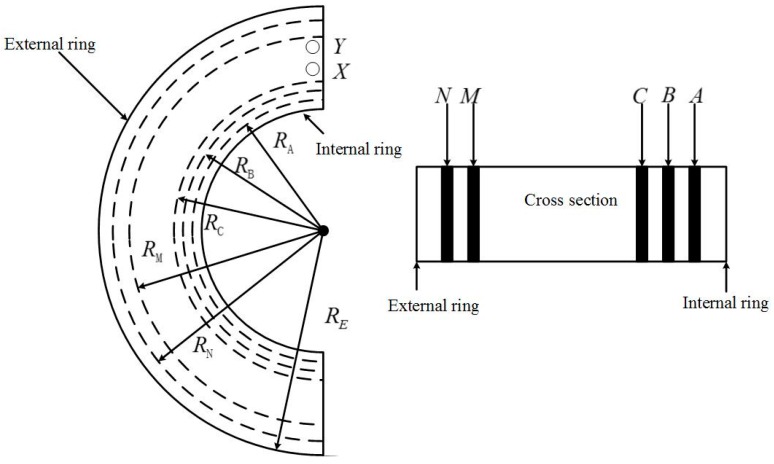
Structure of rows of vias.

**Figure 7:**
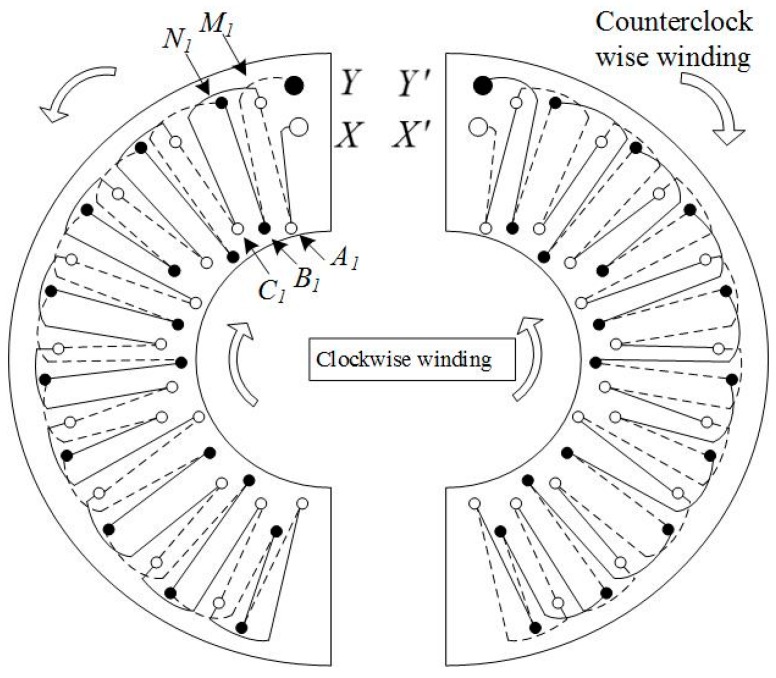
Connections of turns.

As stated before, if the Rogowski coil only has a signal line, the equivalent large turn is vulnerable to vertical magnetic field interference. A return line is indispensable to eliminate this interference. However, the sensor should be an open structure, so how to design the return line on an open structure sensor is a significant problem for fault current detection. Differential winding, which can achieve the same effect as return lines (Figure 6 and Figure 7) is chosen to eliminate this interference. Rows of vias were employed in each semi-ring. The same conductor was folded into two coils that twined on the bobbin towards the same direction in each semi-ring; this process is known as collinear twisted pair differential winding. One coil was defined as clockwise winding, and the other was counterclockwise winding. Given that the currents in these two coils flow in the opposite direction, a 180° difference exists in their phase position. Two coils were wound tightly in a twisted pair to ensure that the branch and return line were strictly symmetric. The generated coupling magnetic fluxes had the same absolute value but opposite directions. They offset each other, thus neutralizing the common-mode signal and eliminating vertical magnetic field interference.

Two rows of vias were set on one side of the external ring of the semicircular printed board, and three rows of vias were set on one side of the internal ring. They were marked from external to internal as Rows N, M, C, B and A. The distances between these rows of vias and the circle center were recorded as *R_N_*, *R_M_*, *R_C_*, *R_B_* and *R_A_*. One end of the semi-ring comprised the starting via X and end via Y of the turn. External Rows N and M had 58 vias, which were uniformly distributed at an equivalent radian interval of π/58. Internal Rows C, B, and A had 39 vias, which were uniformly distributed at an equivalent radian interval of π/39. The copper foil connections of turns in this design are shown in Figure 7.

In Figure 7, the full lines are front connections and the dotted lines are back connections. The hollow dots are the vias on the clockwise winding, and the solid dots are the vias on the counterclockwise winding. The clockwise winding and counterclockwise winding form one differential line pair. The current in the counterclockwise winding was the complementary signal on the clockwise winding. The left semi-ring was taken as an example. The vias from top to bottom were marked as Np, Mp, Cq, Bq, and Aq, where p ranged from 1 to 58 and q ranged from 1 to 39. In this way, the clockwise winding can be expressed as X-A1-M1-C1-M2-B2-M3-A3-M4-C3, …, and the vias of Row M were connected with Rows A, B, and C successively until M58 was connected with C39. On the backboard, C30 was connected with N58 and counterclockwise winding began at N58. The counterclockwise winding was from bottom to top, but the turns remained in the same direction as the clockwise winding. The counterclockwise winding was N58-B39-N57-C39-N56-A38, …, and the vias of Row N were connected with those of Rows A, B, and C successively until B1 was connected with Y. When the front Row N was connected with Rows A, B, and C, it reached across the external side of Row M along a part of small arc. When Back Row M was connected with Rows A, B, and C, it also reached across the external side of Row N along a part of a small arc. The front and back arcs had an equal radius, and the total span was equal. The entire coil was composed of two completely identical semi-rings rather than two mirrored semi-rings. The left semi-ring can be the right one after flipping horizontally. The front wiring of the left semi-ring was the same as the back wiring of the right semi-ring. As a result, two pieces of signal lines are twisted back and forth, thus making *R_re_* and *R* strictly equal. However, the signal phase were completely opposite. The induced electromotive forces produced by the vertical disturbing magnetic field in the equivalent large turn of clockwise winding and the equivalent large turn of counterclockwise winding had the same absolute value but opposite direction. They offset each other, thus, the disturbance of the vertical disturbing magnetic field was eliminated.

### 4.3. Parameters of the Rogowski Coil

**Figure 8:**
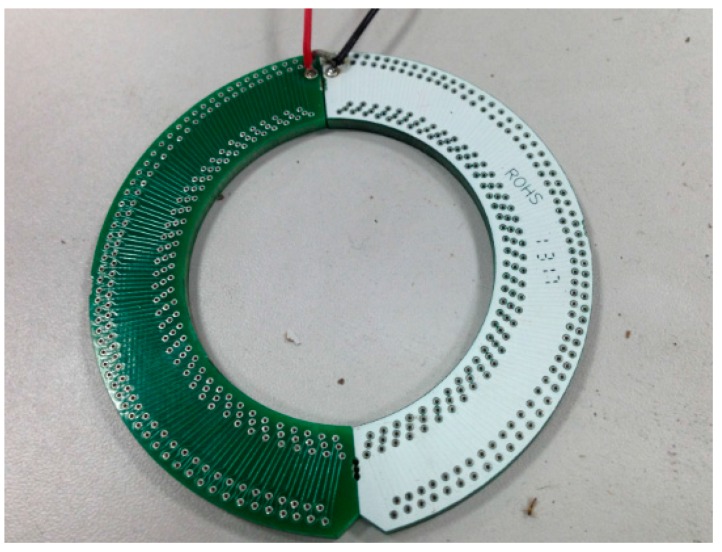
Physical picture of the designed Rogowski coil.

Figure 8 illustrates a physical Rogowski coil composed of two semi-rings. The geometric parameters are presented in Table 2.

Three rows of internal vias of the PCB coil were successively connected to two rows of external vias to form six specifications of small turns (Table 3).

The connection between Y and Y′ was identified as turn type 5, *i.e.*, the turn formed by NB. The total mutual inductance [20] of the coil was the sum of the mutual inductance of different types of turns:

(10) $$M=\sum_{i=1}^{i=6}M_{i}=\sum_{i=1}^{6}N_{i}\frac{\mu_{0}h}{2\pi }\text{ln}\frac{R_{Oi}}{R_{Ii}}$$

where *M_i_* is the mutual inductance of the *i*th type of small turn; *N_i_* is the number of turns of the *i*th type of small turn; *R_Oi_* and *R_Ii_* are the outer radius and inner radius of the *i*th type of small turn, respectively. The structure of multiple rows of vias realized counterclockwise winding of coil and made effective use of the space on the PCB. The self-inductance can be expressed as follows:

(11) $$L_{C}=\sum_{i=1}^{i=6}N_{i}M_{i}=\sum_{i=1}^{6}N_{i}^{2}\frac{\mu_{0}h}{2\pi }\text{ln}\frac{R_{Oi}}{R_{Ii}}$$

**Figure 9:**
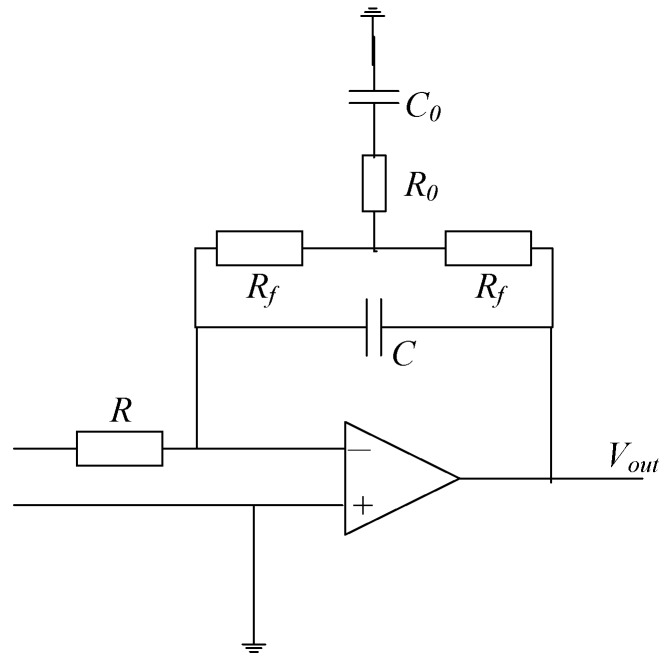
The schematic diagram of integrator.

The main frequency component of the fault current ranges from 50 Hz to several hundred kHz, so the work frequency band of the sensor should cover most of the frequency band. A T-integrating circuit was designed for the PCB Rogowski coil as shown in Figure 9.

The transfer function of Rogowski coil can be obtained from Figure 1b, so the total transfer function of the sensor was [21,22]:

(12) $$H\left(s\right)=\frac{V_{out}\left(s\right)}{I_{1}\left(s\right)}=K_{0}\cdot \frac{T_{0}s+1}{{\left(Ts+1\right)}^{2}}\cdot \frac{K_{c}Ms}{T_{c}^{2}s^{2}+2\xi T_{c}s+1}$$

(13) $$K_{0}=\frac{2R_{f}}{R},T_{0}=\left(\frac{1}{2}R_{f}+R_{0}\right)C_{0},T=R_{f}C+\frac{1}{2}R_{0}C_{0},K_{c}=\frac{R_{s}}{R_{c}+R_{s}},T_{c}=\sqrt{K_{c}L_{c}C_{c}},\xi =\frac{L_{c}+R_{c}R_{s}C_{c}}{2\sqrt{R\left(R_{c}+R_{s}\right)L_{c}C_{c}}}$$

**Figure 10:**
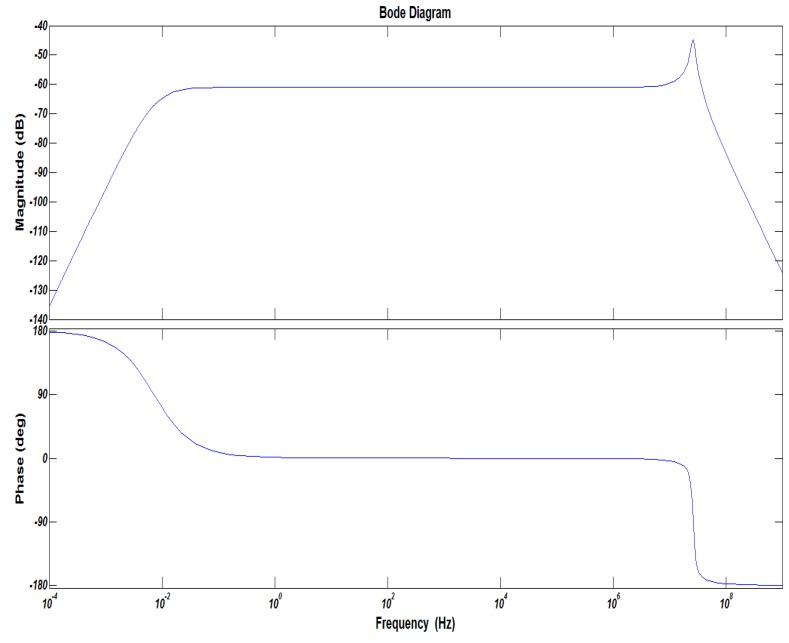
The frequency response of the proposed sensor.

If the appropriate parameters of *R_f_*, *R_s_*, *R*, *C* and *C*_0_ are selected, the corresponding performance can be obtained. If the work frequency bandwidth of the sensor was 30 Hz to 1 MHz, the parameters of the integrator are listed in Table 4, and the corresponding frequency response is shown in Figure 10.

## 5. Performance Test the PCB Rogowski Coil

In order to verify the performance of the proposed sensor, a serial test, including the frequency response, linearity, anti-interference performance as well as a comparison with commercial sensors were conducted. 

### 5.1. Linearity Test

**Figure 11:**
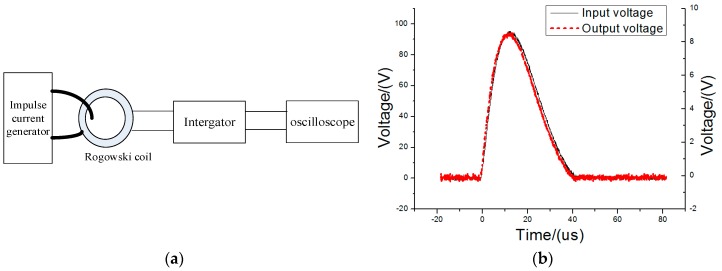
The verification of the PCB Rogowski Coil. (**a**) Experimental linearity circuit; (**b**) Waveform comparison between the input and PCB coil output.

An experimental platform was established to test the high-frequency transient current measurement accuracy of the proposed PCB Rogowski coil (Figure 11a). The impulse current generator can produce 8–20 µs impulse currents to a peak value 50 kA. The output of the impulse current generator and the output of the PCB Rogowski coil were connected onto Channels 1 and 2 of a DPO 5204 digital oscilloscope, respectively. When the impulse current is 10,160 A, the output of the PCB coil is presented in Figure 11b. Figure 11b shows that the output of the PCB coil is almost the same as the input waveform, so the PCB coil has a sufficient bandwidth to detect the fault current.

**Figure 12:**
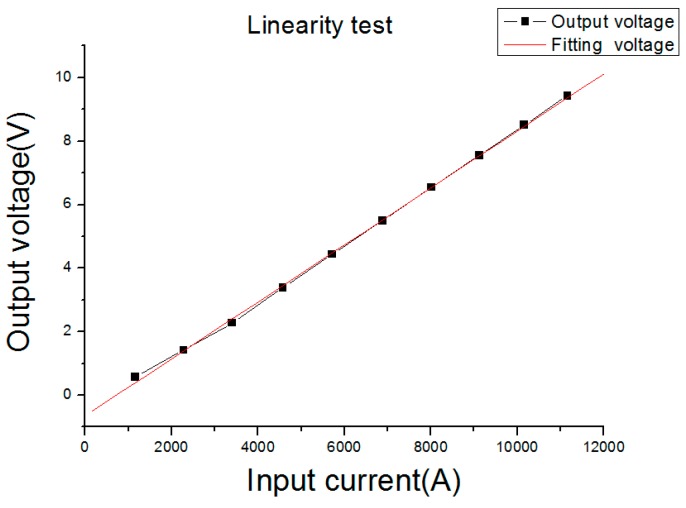
Linearity test fitting curve.

Figure 12 shows the output voltage of the PCB Rogowski coil under different input currents. From Figure 12, the linearity of the PCB Rogowski coil was no more than 2% under a 8–20 µs lightning impulse wave in a 12,000 A range.

### 5.2. Test of Adjacent Wire Interference

**Figure 13:**
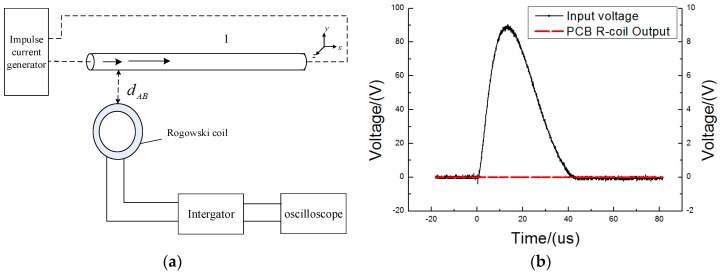
The test of adjacent wire interference. (**a**) Testing diagram of adjacent wire interference; (**b**) The waveform comparison at 2002 A and 3 cm.

The output line of the impulse current generator was extended to simulate the effect of adjacent wires on the PCB Rogowski coil. The plane where the PCB Rogowski coil was located was perpendicular to the wire (Figure 13a). The amplitude of the impulse current generator was set at 2310 A, and the distance between the wire and coil was controlled at 3 and 6 cm. The inputs and outputs of the PCB Rogowski coil were recorded (Figure 13b). The different directions of coil and wire were further changed to test the outputs under different conditions (Table 5).

In Figure 13b, the PCB coil was 3 cm away from the wire, and the coil plane was perpendicular to the wire. When a 2002 A impulse current was set in the adjacent wire, a 96.0 V impulse voltage was generated in Channel 1, while the Rogowski coil output was only 120 mV. Hence, the Rogowski coil with two semi-rings could resist the interference of adjacent wires well.

### 5.3. Test of Vertical Magnetic Field Interference

**Figure 14:**
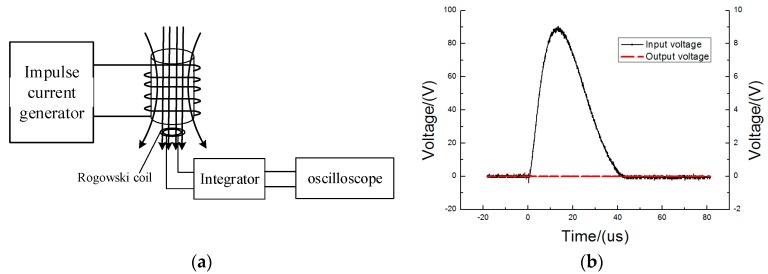
The test of vertical magnetic field interference. (**a**) Testing diagram of vertical magnetic field interference; (**b**) The waveform comparison at vertical magnetic field interference.

The impulse current generator was used to produce an 8/20 interference current waveform and to test the effect of vertical wire on the PCB Rogowski coil. A wire that transmitted the interference current was wound on a cylinder to form a solenoid. This solenoid would generate a uniform magnetic field perpendicular to the horizontal plane, as shown in Figure 14a. The impulse current was set to 1030, 1565 and 2020 A. The vertical disturbing magnetic field was put perpendicular to the PCB Rogowski coil (Figure 14a). The outputs of a conditioning circuit were measured (Table 6).

According to Table 6 and Figure 14b, when the interference current increased from 1030 A to 2000 A, the output of the Rogowski coil was about 450 mV. Thus, the designed PCB Rogowski coil had good resistance to the vertical magnetic field interference.

### 5.4. Test of Frequency Response 

**Figure 15:**
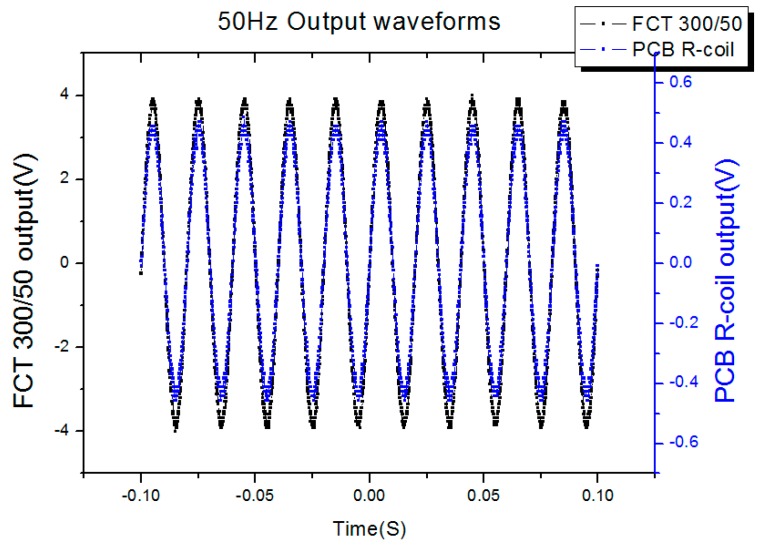
50 Hz output waveforms.

**Figure 16:**
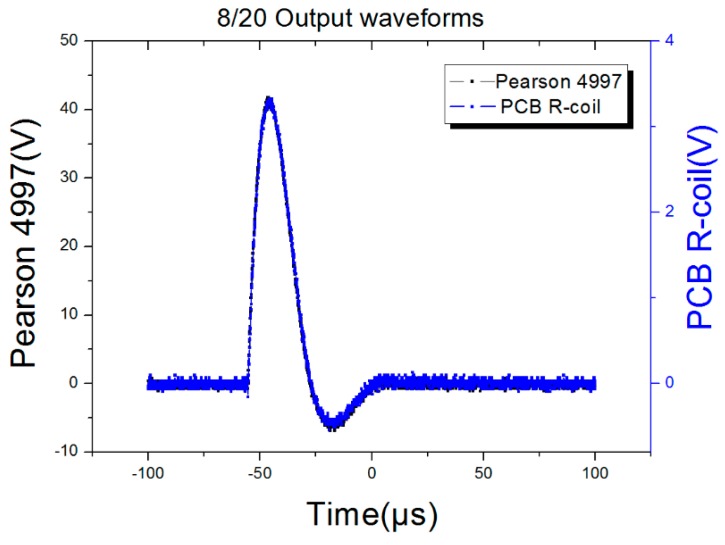
8/20 impulse current output waveforms.

**Figure 17:**
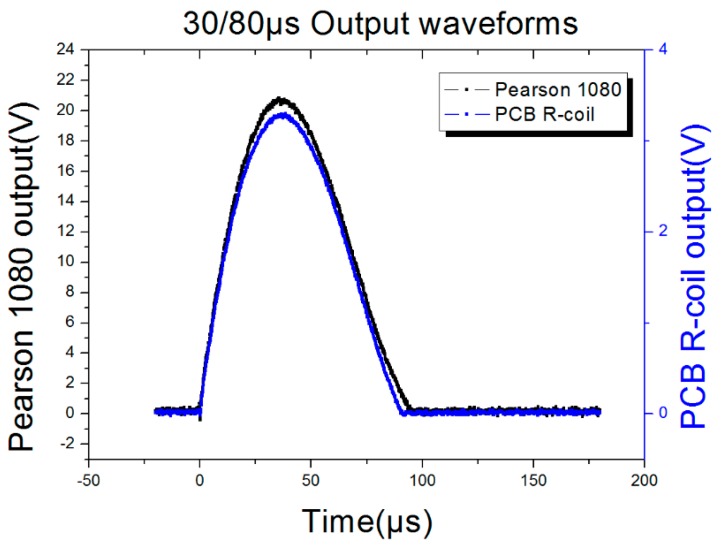
30/80 impulse current output waveforms.

In order to test the frequency response of the proposed sensor, 50 Hz, 8/20 μs and 30/80 μs currents were selected as the current source, so the frequency could cover 50 Hz to 43.75 kHz. The parameters of the current source were listed in Table 7 and the test results were listed in Table 8, while the output waveforms of the PCB Rogowski coil with different source were listed in Figure 15, Figure 16 and Figure 17. The output waveforms are almost the same with the standard current source, meanwhile, the input, the output ratio is 1185:1, 1197:1 and 1208:1, corresponding to the 50 Hz, 8/20 μs and 30/80 μs, respectively, so the work frequency bandwidth covers the bandwidth of the three sources. 

### 5.5. Performance Comparation Between the Commecial Sensors

In order to compare the performance among the newly designed sensor and commercial ones, two commercial sensors, a Pearson 4997 and a FCT 200 were selected. Their main specifications are shown in Table 9. 

**Figure 18:**
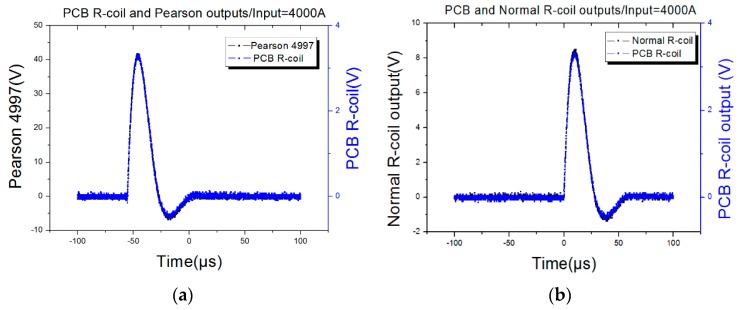
Output waveforms of 8–20 µs impulse current. (**a**) PCB R-coil and Pearson output waveforms; (**b**) PCB and Normal R-coil output waveforms.

After setting the input current range from 1000 A to 5000 A, and recording the output of different sensors, Figure 18 shows that when the input current was 4000 A, the output waveform of the proposed sensor was almost the same as the output of the Pearson 4997 and FCT 200 but for the output ratio. If the Pearson 4997 is considered as the standard, the outputs among the three sensors are listed in Table 10. From Table 10, the linear correlation coefficient of the PCB Rogowski coil is 0.99959, and that of the FCT 200 is 0.99969, so there is nearly no difference between the proposed sensor and commercial ones.

## 6. Field Application

**Figure 19:**
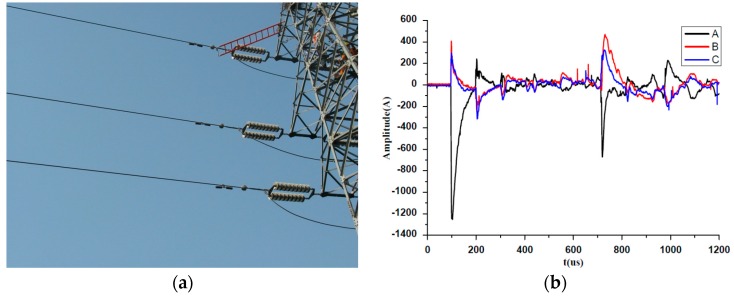
The field application of the proposed sensor. (**a**) Locators installed in field; (**b**) The captured fault current waveforms.

The newly designed sensor has been implemented in an overhead transmission line online monitoring device named distribution fault locator as shown in Figure 19. The low frequency components under 1 kHz were eliminated by a high pass filter because only the high frequency was of concern in the application. 

Figure 19 shows the amplitude of the captured fault travelling wave current was 1254 A, the half wavelength was about 26 μs, while the rise time was about 5 μs, which was fully adequate for transient fault current detection, so the designed PCB sensor has excellent performance, as good as that of the commercial ones.

## 7. Conclusions

An open structure PCB Rogowski coil has been presented. The mechanism of interference around the overhead transmission line and the guidelines of anti-interference were established. The proposed PCB Rogowski coil according to the guidelines was designed and tested. For transient fault current detection, the proposed PCB Rogowski coil has almost the same excellent performance in work frequency bandwidth and linearity as commercial ones. Furthermore, the strictly symmetrical structure of the PCB Rogowski coil could eliminate almost all the magnetic field interference from the radial direction, so it is immune from the interference of adjacent conductors, while, the differential winding of the PCB Rogowski coil could offset the axial magnetic field interference, and as a result, the proposed sensor has perfect anti-interference performance, which was the key to measuring transient fault currents. Through the tests, the proposed PCB Rogowski coil was proved to be more machinable, repeatable, and of commercial value. 

## Figures and Tables

**Table 1:** Coefficients of (linear) thermal expansion of common bobbin materials.

Material	Rubber	Polyethylene	Epoxy Resin	Ceramic
Coefficient of expansion/(10^−6^/°C)	200–300	126–160	60–80	1.0–7.7

**Table 2:** Geometric parameters of the PCB Rogowski coil.

Inner Radius of the Bobbin	Outer Radius of the Bobbin	Thickness	RA	RB	RC	RM	RN
24.0 mm	39.0 mm	3.0 mm	25.5 mm	26.5 mm	27.5 mm	36.5 mm	38.0 mm

**Table 3:** Specifications of small turns.

Turn Type	1	2	3	4	5	6
Outer Row	M	M	M	N	N	N
Outer Row	A	B	C	A	B	C
Turns	38	38	40	38	38	40
Inner radius (mm)	25.5	26.5	27.5	25.5	26.5	27.5
Outer radius (mm)	36.5	36.5	36.5	38.0	38.0	38.0

**Table 4:** Parameters of the PCB Rogowski Coil sensor.

Parameters	Values	Parameters	Values	Parameters	Values
*R_C_*	46.3 Ω	*M*	47.346 nH	*R*_0_	2 MΩ
*L_C_*	1.8283 µH	*R_s_*	20 MΩ	*C*_0_	22 µF
*C_C_*	20.12 pF	*R_f_*	1 kΩ	*C*	1 µF

**Table 5:** Interference test of adjacent wire.

Coil Flat Position	*xz*		*xy*		*yz*	
*d_AB_* (cm)	3	6	3	6	3	6
Impulse current (A)	1969	1969	1901	1901	2002	2002
Peak value of channel 1 (V)	94.4	94.4	91.2	91.2	96	96
Peak value of channel 2 (mV)	220	220	140	140	120	160
Error%	0.233	0.233	0.153	0.153	0.125	0.167

**Table 6:** Test results of vertical magnetic field interference.

Impulse current (A)	1030	1565	2020
Peak value of channel 1 (V)	48	64	94
Peak value of channel 2 (mV)	420	450	480
Error%	0.875	0.7	0.51

**Table 7:** Models and equipment parameters.

Parameters	Source 1	Source 2	Source 3
Current parameters	50 Hz	8/20 (μs)	30/80 (μs)
Source models	PY002	KV2103-G-020	MWG001
Standard CT	FCT 300/50 150 A/V	Pearson 4997 100 A/V	Pearson 1080 200 A/V

**Table 8:** Results of the frequency experiments.

Current Waveforms	50 Hz	8/20 (us)	30/80 (us)
Standard CT outputs (V)	2.77	41.42	20.78
Input current (A)	415	4142	4156
PCB R-coil outputs (V)	0.35	3.46	3.44
PCB R-coil ratio	1185:1	1197:1	1208:1

**Table 9:** The main specifications of two commercial sensors.

Sensor	Pearson Model 4997	Normal Rogowski Coil FCT 200
I/O ratio	100 A/1V	500 A/1V
Bandwidth (−3 dB)	0.5 Hz–15 MHz	1 Hz–1 MHz
Accuracy	1%	1%

**Table 10:** Outputs of different sensors.

No.	Setting Input Current (A)	Pearson 4997 Output (V)	PCB R-Coil Output (V)	FCT 200 Output (V)
**1**	1000	12.0	1.00	2.54
**2**	2000	23.3	1.98	4.62
**3**	3000	31.9	2.66	6.52
**4**	4000	41.8	3.46	8.49
**5**	5000	51.7	4.38	10.41
